# Dapagliflozin cardiovascular effects on end-stage kidney disease (DARE-ESKD-2) trial: rationale and design

**DOI:** 10.21203/rs.3.rs-3434207/v1

**Published:** 2023-10-19

**Authors:** Joaquim Barreto, Marilia Martins, Mauro Pascoa, Sheila T. K Medorima, Isabella Bonilha, Daniel Campos Jesus, Cinthia E. M. Carbonara, Kelcia R. S. Quadros, Barbara Assato, Alessandra M Campos-Staffico, Gil Guerra Júnior, Wilson Nadruz, Rodrigo B. de Oliveira, Andrei C Sposito

**Affiliations:** UNICAMP: Universidade Estadual de Campinas; UNICAMP: Universidade Estadual de Campinas; UNICAMP: Universidade Estadual de Campinas; UNICAMP: Universidade Estadual de Campinas; UNICAMP: Universidade Estadual de Campinas; UNICAMP: Universidade Estadual de Campinas; UNICAMP: Universidade Estadual de Campinas; UNICAMP: Universidade Estadual de Campinas; UNICAMP: Universidade Estadual de Campinas; Creighton University; UNICAMP: Universidade Estadual de Campinas; UNICAMP: Universidade Estadual de Campinas; UNICAMP: Universidade Estadual de Campinas; UNICAMP: Universidade Estadual de Campinas

## Abstract

**Purpose.:**

Sodium glucose co-transporter 2 inhibitors (SGLT2i) remarkably reduced the incidence of hospitalization for heart failure and cardiovascular death of conservatively managed chronic kidney disease. We hypothesized that adding SGLT2i to standard treatment would yield cardiovascular benefits also in end-stage kidney disease (ESKD) individuals on dialysis.

**Methods.:**

The DARE-ESKD-2 Trial (NCT05685394) is an ongoing, single-center, open-label, controlled trial aimed at assessing the cardiovascular effects of dapagliflozin in ESKD on dialysis. Eligible patients are adults on renal replacement therapy for more than 3 prior to enrollment. Exclusion criteria encompass pregnancy, liver failure, and current use of a SGLT2i. After signing an informed consent form, participants are randomized 1:1 to either dapagliflozin 10mg PO plus standard treatment or standard treatment alone for 6 months. Echocardiogram, anthropometry, blood sample collection, 6-min walk test, gait speed, and Kansas City Cardiomyopathy Questionnaire (KCCQ), are performed at baseline and at study termination. Participants are contacted monthly during treatment for outcomes disclosure. The primary endpoint of our study is the between-groups differences in posttreatment changes in plasma levels of N-terminal pro-B natriuretic peptide. Secondary endpoints include the differences between groups in the changes of echocardiography measurements, cardiopulmonary tests performance, body composition. The incidence of safety endpoints will also be diligently compared between study arms.

**Conclusion.:**

The DARE-ESKD-2 trial will provide unprecedented data on the cardiovascular safety and efficacy of SGLT2i in ESKD individuals on dialysis. This study will pave the grounds for improving clinical outcomes of dialysis recipients.

## INTRODUCTION

Current estimates project a troubling scenario: the prevalence of end-stage kidney disease (ESKD) requiring renal replacement therapy (RRT) is expected to soar to a staggering 15 million before 2030.^1^ This figure is particularly alarming when we consider that these patients face a 20-fold higher risk of major cardiovascular events compared to individuals with preserved renal function. These events currently account for six out of every ten deaths within the already concerning 20% annual mortality rate associated with RRT.^2^ This grim reality stems from a well-stablished cardiorenal interplay, where deteriorating kidney function triggers a cascade of multisystem organ dysfunction. This chain reaction ultimately leads to heart failure, disautonomia, hypertension, and atherogenesis, all of which contribute to the substantial cardiovascular burden experienced by those undergoing dialysis.^2,3^

Despite these statistics, individuals with ESKD have been excluded from landmark clinical trials investigating inhibitors of the sodium glucose cotransporter 2 (SGLT2i), which consistently demonstrated significant cardiorenal protection.^4^ In fact, both in DAPA-CKD and EMPA-KIDNEY trials, SGLT2i treatment led to a remarkable one-third reduction in the incidence of a composite outcome comprising cardiovascular death and heart failure hospitalization among individuals with chronic kidney disease (CKD) stages 3 to 4.^5,6^ From the mechanistic perspective, these benefits were primarily attributed to natriuresis and glycosuria, which contributed to improved volume and glycemic controls.^7^ Nevertheless, as our understanding has deepened, evolving evidence has unveiled a plethora of other advantageous effects associated with SGLT2i treatment. These effects extend beyond kidney function and encompass enhancements in endothelial function, myocardial energy metabolism, and the mitigation of dysautonomia.^8^ Consequently, SGLT2i emerges as a promising therapeutical target for preventing cardiovascular morbidity, even in individuals with severely impaired kidney function who require dialysis.^9^

In this scenario, having outlined the safety profile and pharmacokinetics of dapagliflozin in dialysis, we hereby present the study design for the DARE-ESKD-2 trial, which aims to explore the cardiovascular effects of dapagliflozin in ESKD.^10^ The primary objective of this study is to investigate whether dapagliflozin influences cardiac remodeling markers. Secondarily, this study aims to explore its potential role also on cardiopulmonary performance, quality of life, adiposity, and bone metabolism. The data gleaned from this study could also serve as a foundation for future mechanistic hypothesis generation and assessments aimed at long-term prevention of major cardiovascular events.

## METHODS

### Study design and population

The DARE-ESKD-2 study (NCT05685394) is an ongoing randomized, phase 3, controlled, open-label trial aimed at assessing the cardiovascular effects of dapagliflozin vs. standard treatment in individuals with ESKD who are undergoing dialysis. The study protocol received ethical approval from the local ethics committee, and it complies with the principles outlined in the Declaration of Helsinki.^11^ Eligible participants are adults aged 18 years or older who have been on regular hemodialysis or peritoneal dialysis for at least three months prior to enrollment. Exclusion criteria encompass hypersensitivity to any components of the study drug, pregnancy, impaired liver function, recent use of SGLT2i, or experiencing acute coronary syndrome within the three months preceding randomization.

### Clinical care protocol

**Figure 1:**
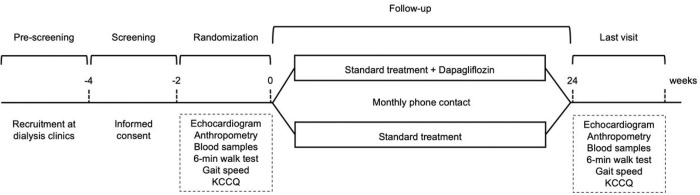
Study protocol.

The study protocol, outlined in Fig. 1, begins with patient referrals from local dialysis clinics to our research center, where undergo their initial medical assessment to determine eligibility. During this visit, a certified physician presents the informed consent form and conducts a thorough interview to gather medical history register, followed by a comprehensive physical examination. Then, participants are randomly assigned in a 1:1 ratio either dapagliflozin (10mg/day) plus standard treatment or standard treatment alone (control group) using an automated system.

Furthermore, peripheral blood samples are collected for biochemical analysis, and a 12-lead electrocardiogram is obtained. Blood pressure readings are taken in triplicate after 3 minutes of rest in a seated position and repeated after 1 minute of standing to assess orthostatic changes, using the Omron Hbp-1120 device (Omron Healthcare, Japan). Additionally, assessments through dual X-ray absorptiometry (DXA), bioimpedance, echocardiography, gait speed measurement, and six-minute walk test are performed during this visit.

Once the baseline evaluation is completed, patients in the dapagliflozin group receive the necessary pills for a 24-week treatment period, and all participants are thereupon contacted monthly to report outcomes and monitor medical adherence until study’s conclusion, at which baseline evaluations are repeated.

### Echocardiography

Transthoracic echocardiogram is performed by experienced cardiologists blinded to patients allocation and comply with the latest recommendation from the American Society of Echocardiography.^12^ Heart ultrasound images are obtained by an equipment with three-dimensional (3D) technology (Philips EPIQ CVX ultrasound system, Philips Medical Systems, Andover, MA, USA) and a transducer for real time multiplanar volumetric analysis (X5–1 xMATRIX array transducer with PureWave crystal technology, 3040 elements, 5 – 1 MHz). Cardiac chambers diameters, volumes, indexed left ventricle (LV) mass estimation and LV ejection fraction are analyzed by both 2D and 3D methods (*HeartModel*^A.I^., Philips Medical Systems, Andover, MA, USA).

Complete echocardiographic analysis included appearance of the thoracic aorta, analysis of cardiac flows and presence of valvular heart disease. Diastolic function analysis followed the latest guidelines and included the E/e’ ratio, calculated from mitral inflow E velocity and mean tissue Doppler myocardial e’ velocity from mitral annulus (septal, lateral, anterior, and inferior) as an indirect estimation of the left atrium pressure. Global longitudinal strain by speckle tracking used a dedicated automated tool (Automated Cardiac Motion Quantification^A.I^., aCMQ^A.I^.,Philips Medical Systems, Andover, MA, USA).^13,14^

### Body Composition

Body composition is evaluated using a DXA device (Lunar iDXA enCore version 13.60, GE Healthcare, USA) administered by an accredited anthropometrist who is blinded to participant allocation. Image acquisition and standardization of region of interest comply with the latest guidelines set forth by the International Society for Clinical Densitometry. The equipment undergoes weekly calibration for accuracy.^15^ This examination yields data on lean and fat mass as well as bone mineral density with the respective scores at specific anatomical sites including the femoral neck, Ward’s triangle, trochanter, and lumbar spine.. In case where patient find it challenging to fit into the equipment, an image mirroring technique is employed.^16^

During the same assessment, we utilize bioimpedance analysis (Model-450, Biodynamics Corporation, Seattle, WA, USA) to obtain information on resistance, reactance, estimated basal metabolic rate, fat mass percentage, lean mass, body water, and phase angle. These measurements are analyzed inconsideration of body mass, age, and gender.^17^ As part of our comprehensive evaluation, we also obtain data on weight, height, and arm circumference according to validated protocols.^18,19^

#### Cardiopulmonary functional testing

The 6-minutes’ walk test (6MWT) adheres to the guidelines set forth by the American Thoracic Society.^20^ Briefly, participants are instructed to maintain their swiftest walking pace without running in a demarcated corridor for precisely 6 minutes, with supervision provided by a researcher responsible for registering the distance traversed.^4^ Readings for pulse oximetry, blood pressure, and heart rate are registered prior to commencing the test and immediately after it is terminated. Following the conclusion of the test, patients convey their perceived effort level using the Borg Scale.^4^ The 6MWT is contraindicated for patients with a resting heart rate exceeding 120 beats per minute, or a systolic or diastolic blood pressure exceeding 180mmHg and 100mmHg, respectively. Subsequently, patients are instructed to walk at their maximum pace within the same corridor, while the evaluator register the time taken to cover a precisely marked 4-meter distance. This evaluation is conducted three times, and the best time out of the three trials is utilized for analyzing 4-meter gait speed.

Additionally, handgrip strength is assessed in triplicate using a hand dynamometer (SH5001, Saehan Corporation, Dangjin-gun, South Korea), and the best result out of the three measurements (in Kg) is employed in our analysis.^21^

### Kansas City Cardiomyopathy Questionnaire

The Kansas City Cardiomyopathy Questionnaire (KCCQ) comprises a 23 self-administered questions, addressing a wide range of physical, psychological, and social symptoms that may arise from heart failure.^22^ The KCCQ yields scores on a scale of 0 to 100 points, where higher scores correspond to a better quality of life.^22^ Study participants fill the Portuguese-validated KCCQ prior to randomization and at the study conclusion.^23^

### Biochemical analysis

Peripheral blood samples are collected and dispatched to the clinical analysis laboratory for a comprehensive panel of tests. These tests include a complete blood count (flow cytometry and impedance – DXH 800), urea (urease with GLDH), creatinine (kinetic – IFCC), sodium, calcium, phosphate, and potassium (Selective Electrode), venous blood gases (Sensor-OMNI), aspartate aminotransferase (AST) and alanine aminotransferase (ALT) (kinetic – IFCC), and alkaline phosphatase (Abcam, Cambridge, UK).

To facilitate enzyme immunoassays, blood samples are collected and centrifuged at 3,500 rpm for 10 minutes. Plasma aliquots are cryopreserved in liquid nitrogen for subsequent measurements of N-terminal pro-B natriuretic peptide (NT-proBNP, Human pro-BNP Georgia, USA), fibroblast growth factor-23 (FGF-23; Abcam, Cambridge, UK), and parathyroid hormone (Sigma-Aldrich, San Luis, Missouri, EUA), α-Klotho (Quidel, San Diego, USA), and MILLIPLEX MAP Human Bone Magnetic Bead Panel - Bone Metabolism Multiplex Assay (Merck, Darmstadt, Germany). These quantitative measurements are performed in accordance with the manufacturer’s recommendations, and the absorbance is determined using a microplate reader (Biotek EPOCH 2).

### Follow-up

Following randomization, participants receive monthly phone calls over a six-month period. During each contact, they are queried about various aspects, including changes in their dry weight, alterations in bowel and urinary habits, their overall well-being during daily activities, blood pressure measurements, occurrences of fractures, any medical procedures undergone, initiation of new treatments, surgeries, hospitalizations, infections, and clinical incidents. Additionally, participants in the dapagliflozin group are required to report the number of study drug pills taken in the past 30 days to track medication adherence, which is registered as a percentage.

### Efficacy endpoints

The primary endpoint of our study is the between-groups differences in posttreatment changes in plasma levels of NT-proBNP. Secondary endpoints encompass the differences between groups in the changes of LV mass, EF, E/e’ ratio, KCCQ points, 6-min walk test distance, gait speed, lean to fat mass ratio, and handgrip strength.

In addition, an exploratory analysis will be conducted, in which participants with residual diuresis will be inquired about changes in their urinary output. Furthermore, the incidence of a composite outcome comprising cardiovascular death, hospitalization for heart failure, and stroke will be compared between the dapagliflozin and control groups. Changes in serum levels of bone metabolism markers, such as FGF-23 and a-Klotho, as well as on bone mineral density, will be compared between groups as an exploratory analysis.

#### Safety endpoints

For safety assessments, we are diligently monitoring adverse events at each visit, following the guidelines outlined in the Patient Reported Outcomes version of the Common Terminology Criteria for Adverse Events from the National Cancer Institute^24,25^. Safety Endpoints in this clinical trial encompass the following: (i) Time to the first occurrence of the adverse events (AE): (a) Liver injury, evaluated both overall and individually by causative factors (defined as ALT or AST levels ≥ 5x Upper Limit of Normal [ULN], or the combination of ALT or AST ≥ 3x ULN with bilirubin ≥ 2x ULN measured in the same blood sample); (b) Ketoacidosis, assessed both overall† and categorized by baseline diabetes status; (c) Lower limb amputations, considered overall† and differentiated by level (toe, forefoot, foot, below knee, or above knee); (ii) Time to the first occurrence of another AE relevant to the study’s objective: (a) Bone fracture, examined both overall and categorized by site (long bones versus non-long bones) and etiology (distinguishing those resulting from high and low impact trauma, and other causes); (b) Symptomatic dehydration, defined by whether participants experience symptoms attributable to dehydration, such as feeling faint or fainting; (iii) Time to the first occurrence of hospitalization, categorized by specific causes according to the MedDRA Primary System Organ Class (SOC); (iv) Time to the first occurrence of Serious Adverse Events (SAEs), both overall† and separately, by Primary SOC category; (v) Discontinuation of study treatment, assessed overall and categorized by various causes, including SAEs, non-serious adverse events, and other reasons, using Primary SOC categories for SAEs and non-serious adverse events.

### Power and sample size

Based on previous trials involving dapagliflozin, the sample size was meticulously calculated, considering an expected between-groups difference of 9% in the primary endpoint of the relative change of NT-proBNP. With a significance level (alpha). Set at 0.05 and a statistical power (beta) of 80%, the calculation indicated a requirement of 26 patients in each arm, totaling 52 participants. However, in anticipation of potential dropouts and with consideration for secondary outcomes, researchers opted for a more robust sample size of 80 patients. The calculations were performed using G*Power 3.1 Mac version (Heinrich-Heine-Universität, Germany).

### Data management

Data management in our study relies on the utilization of the Research Electronic Data Capture tool (REDCap, Nashville, TN), which is a web-based and secure platform. REDCap serves the critical function of facilitating the auditing of data collection processes and the seamless export of data. This ensures the creation of de-identified datasets, which is a paramount for safeguarding the confidentiality of our study participants.^26^ The principal investigator assumes the pivotal role of compiling a comprehensive summary of examination results, which is the distributed to all participants involved in the study. Additionally, the principal investigator is responsible for delineating the specific roles of sub-investigators within the research team.

### Statistical analysis

Unless specified otherwise, all analyses will entail an intention-to-treat comparison involving all randomized participants. The groups will be compared in terms of their differences from baseline using analysis of covariance (ANCOVA) or rank analysis of covariance (RANKOVA) according to the data distribution and considering baseline values and study arm as covariates. Continuous variables will be expressed as mean ± standard deviation (SD) for normally distributed, or as median (interquartile range) for non-normally distributed data. Categorical data will be presented as counts and percentages. We will employ Student’s t and Mann-Whitney tests to compare means and medians between groups, respectively, while categorical data will be assessed using the chi-square test. Continuous variables may undergo ranking or log-transformation as needed to achieve normality. Adjustments by covariates will be made when appropriate. A statistical significance will be defined as a p-value less than 0.05. The statistical analysis will be carried out using SPSS version 28.00 for Mac, and it will be performed by an accredited statistician.

## DISCUSSION

The DARE-ESKD-2 trial promises to yield groundbreaking insights into the cardiovascular impact of dapagliflozin in patients with ESKD. Building upon the findings of our prior trial, which firstly investigated the pharmacokinetics and safety profile of this drug in individuals undergoing hemodialysis and peritoneal dialysis, our current study aims to uncover the potential cardiovascular benefits of SGLT2i in ESKD.^10^

One significant factor in cardiovascular health among dialysis receivers is the fluid overload on the heart, resulting in abnormal myocardial stretching prompting higher levels of circulating natriuretic peptides (NP).^27^ In a large meta-analysis of 19,000 patients receiving renal replacement therapy (RRT), those in the highest quintile of N-terminal pro–B-type NP (NT-ProBNP) levels experienced a six-fold increase in cardiovascular mortality and a three-fold higher all-cause mortality compared to those in the lowest quintile.^28^ Furthermore, the annual risk of sudden cardiac death increases 5% per each 1ng/mL rise in NT-ProBNP levels. Notably, NT-ProBNP levels also inversely related to indexed left ventricle (LV) mass and diastolic dysfunction grade in ESKD.^28,29^ Additionally, the extent to which NT-ProBNP levels are reduced following an intervention is markedly related to the prevention of cardiovascular death and heart failure-related hospitalization, which are pivotal factors contributing to RRT-related mortality.^30–32^ Hence, the primary outcome of this trial will be difference in NT-proBNP change between the groups.

Structural heart disease affects nine out of ten dialysis receivers, exerting with a substantial impact on long-term mortality rates.^2^ Roughly, half of these patients suffer from LV diastolic impairment, one-third experience right ventricle dysfunction, and about 20% progress to heart failure with reduced ejection fraction (HFrEF).^33,34^ In non-RRT patients, treatment with SGLT2i led to a significant reduction in LV end-diastolic volume (LVEDV) and indexed LV mass by 10mL and 5g/m^2^, respectively, while increasing left ventricular ejection fraction (LVEF) by 2%.^35^ These changes mirror the effects of SGLT2i on myocardial energy supply, ketonemia, suppression of cardiomyocyte death pathways, and improvements in myocardial flow reserve and vasomotor responses.^35–39^ However, the influence of SGLT2i on myocardial function and heart structure, as assessed by echocardiography, has yet to be evaluated in dialysis patients, making it an integral part of the exploratory analysis in this trial.

Dialysis significantly impacts the quality of life (QoL) and functional capacity of patients. Those undergoing RRT are more prone to developing anxiety and depression, and their exercise tolerance is diminished compared to age-matched controls.^40,41^ This study will evaluate QoL through the KCCQ score, which previous studies have shown to be positively influenced by dapagliflozin in patients with HFrEF.^42^ Furthermore, we will assess cardiopulmonary performance using measures such as the 6-minute walk test distance, handgrip strength, and gait speed, while accounting for changes in body composition measured by DXA. Each of these measures hoolds significant prognostic value and has been shown to be influenced by SGLT2i treatment, making posttreatment changes a crucial aspect of this trial’s exploratory analysis.^43–45^

Mineral bone disorders (MBD) contribute to cardiovascular disease progression in dialysis recipients.^46^ Phosphate overload elicits intricated downstream effects on calcium metabolisms prompting arterial media and cardiac valves calcification, hyperparathyroidism-induced ventricular hypertrophy and arrythmia, and osteopenia.^46^ Impaired expression of klotho protein plays an imperative role in the progression of MDB, and this event was attenuated by treatment with SGLT2i *in vivo*.^47,48^ SGLT2i- related changes in klotho and other bone metabolism markers will be analyzed, as well as changes in bone mineral density, compared to standard treatment as an exploratory analysis of this trial.^49^

It is important to acknowledge the limitations inherent of this study. Ethical considerations precluded the prior administration of dapagliflozin to dialysis recipients. Consequently, we have decided for the open-label design, recognizing its shortcomings compared to double-blinded trials. To counteract potential biases stemming from this choice, our evaluators, responsible for conducting echocardiography, cardiopulmonary tests, and biochemical analysis are blinded to patients’ allocation. Also, upon completion of the study, an independent statistician will analyze the dataset devoid of any study group information. Of note, previous breakthrough studies in ESKD, such as the ASCEND-D and INNO2VATE trials, have employed similar study designs.^50,51^

In conclusion, the DARE-ESKD-2 will yield unique insights into the cardiovascular impact of dapagliflozin in dialysis receivers, encompassing both functional and structural myocardial parameters. These findings hold the potential to pave the way for improved clinical outcomes among patients with ESKD.
